# Increased Childhood Mortality and Arsenic in Drinking Water in Matlab, Bangladesh: A Population-Based Cohort Study

**DOI:** 10.1371/journal.pone.0055014

**Published:** 2013-01-28

**Authors:** Mahfuzar Rahman, Nazmul Sohel, Mohammad Yunus, Mahbub Elahi Chowdhury, Samar Kumar Hore, Khalequ Zaman, Abbas Bhuiya, Peter Kim Streatfield

**Affiliations:** 1 icddrb, Mohakhali, Dhaka, Bangladesh; 2 Department of Epidemiology, Mailman School of Public Health, Columbia University, New York, New York, United States of America; 3 Department of Clinical Epidemiology and Biostatistics, McMaster University, Hamilton, Ontario, Canada; University of Louisville, United States of America

## Abstract

**Background:**

Arsenic in drinking water was associated with increased risk of all-cause, cancer, and cardiovascular death in adults. However, the extent to which exposure is related to all-cause and deaths from cancer and cardiovascular condition in young age is unknown. Therefore, we prospectively assessed whether long-term and recent arsenic exposures are associated with all-cause and cancer and cardiovascular mortalities in Bangladeshi childhood population.

**Methods and Findings:**

We assembled a cohort of 58406 children aged 5–18 years from the Health and Demographic Surveillance System of icddrb in Bangladesh and followed during 2003–2010. There were 185 non-accidental deaths registered in-about 0.4 million person-years of observation. We calculated hazard ratios for cause-specific death in relation to exposure at baseline (µg/L), time-weighted lifetime average (µg/L) and cumulative concentration (µg-years/L). After adjusting covariates, hazard ratios (HRs) for all-cause childhood deaths comparing lifetime average exposure 10–50.0, 50.1–150.0, 150.1–300.0 and ≥300.1µg/L were 1.37 (95% confidence interval [CI], 0.74–2.57), 1.44 (95% CI, 0.88–2.38), 1.22 (95% CI, 0.75–1.98) and 1.88 (95% CI, 1.14–3.10) respectively. Significant increased risk was also observed for baseline (*P* for trend = 0.023) and cumulative exposure categories (*P* for trend = 0.036). Girls had higher mortality risk compared to boys (HR for girls 1.79, 1.21, 1.64, 2.31; HR for boys 0.52, 0.53, 1.14, 0.99) in relation to baseline exposure. For all cancers and cardiovascular deaths combined, multivariable adjusted HRs amounted to 1.53 (95% CI 0.51–4.57); 1.29 (95% CI 0.43–3.87); 2.18 (95%CI 1.15–4.16) for 10.0–50.0, 50.1–150.0, and ≥150.1, comparing lowest exposure as reference (*P* for trend = 0.009). Adolescents had higher mortality risk compared to children (HRs = 1.53, 95% CI 1.03–2.28 *vs.* HRs = 1.30, 95% CI 0.78–2.17).

**Conclusions:**

Arsenic exposure was associated with substantial increased risk of deaths at young age from all-cause, and cancers and cardiovascular conditions. Girls and adolescents (12–18 years) had higher risk compared to boys and child.

## Introduction

Arsenic (As) is a known human carcinogen in adults as defined by the International Agency for Research on Cancer (IARC) on the basis of a large body of epidemiological evidence [1]. Indeed, adverse health effects of As in drinking water are a major public health concern in Bangladesh and elsewhere where there is unacceptable levels of As in drinking water. Globally, around 100 million people are exposed, and in Bangladesh the problem has been described as the greatest mass poisoning in history [2], where high concentrations of naturally occurring arsenic found in shallow well water are affecting enormous numbers of people exposed through drinking water [3].

Arsenic in drinking water is established to be a major cause of adult mortality [4]. So far, epidemiological studies in Taiwan [5], Bangladesh [6], [7], Chile [8], and Argentina [9], [10] have reported increased adult mortality associated with arsenic exposure. Only a few studies on childhood mortality have been conducted in relation to arsenic exposure [11]–[15]. Therefore, very little is known about the association of As exposure and risk of childhood deaths. More recently, research has suggested that early-life exposure increases adult onset of chronic diseases [16], [17]. Arsenic may have greater impact on children than adults as suggested by The American Academy of Pediatrics (AAP 2003) for its anti-metabolic and carcinogenic properties, affecting organogenesis and organs maturity taking place during childhood.

Icddr,b Matlab field site established a longitudinal Health and Demographic Surveillance System (HDSS) since the mid-1960s covering a large population and subsequently collecting historical assessment of As exposure for individuals [18], making it an ideal location to investigate the long-term health effects of arsenic in drinking water. This is a unique settings in contrast to settings in other countries (such as Argentina, China, India, Taiwan, and the United States), where high arsenic exposures come primarily from wells at population level and not at individual level. Thus, we set a novel approach to provide reliable estimates of any association between varying levels of arsenic exposure and the risk of all-cause childhood mortalities by analyzing data from 58,406 persons aged 5–18 years who were at risk for a total of 0.4 million person-years. The impact of exposure during childhood is largely unknown. Children are likely to be especially susceptible since organ maturation and much functional development takes place during childhood including many internal organs. We also assessed the risk associated with combined cancer and cardiovascular specific deaths in relation to early-life arsenic exposure.

## Methods

### Ethic Statement

All individuals gave written informed consent to participate. An icddrb institutional review committee and the icddrb Ethical Review Committee approved the baseline study. A mitigation program was initiated in collaboration with Bangladesh Rural Advancement Committee (BRAC), Bangladesh [19].

### Study Area and Design

Matlab, is a typical rural area of Bangladesh which is located 55 kilometres to southeast of the capital Dhaka. Matlab has been most affected in the country by tubewell water arsenic contamination with established adverse health consequences [20], [21].

This prospective study was designed based on a previous study [18] that assembled the child population aged 5–18 years on January 1, 2003. We excluded children under 5 years old because our baseline screening involved persons aged 5 years and older. We followed this child population through December 31, 2010. All the childhood deaths prospectively captured by the monthly Health and Demographic Surveillance System (HDSS) household visits during January 2003 to December 2010 were included. Causes of deaths were ascertained by verbal autopsy (VA) [22].

Follow-up time in person-years was calculated as the number of days between the baselines interview and date of death, out migration, or report of being alive on December 31, 2010 whichever came first. Participants with an accident-related cause of death such as road traffic accident, drowning or other accidental deaths or alive were censored.

### Arsenic Exposure Assessment

All well water samples (n = 13286) were analyzed at baseline by Hydride Generation Atomic Absorption Spectrometry (HG-AAS) for determination of baseline individual level arsenic exposure. Historical drinking water sources were also collected. For children, we interviewed the parents or guardians regarding exposure histories since birth. Details of study methodology and results have been published previously [18].

Three exposure categories [baseline, time- weighted lifetime average (average) and cumulative arsenic exposure] were calculated for each individual. Baseline means current water exposure in time of interview. Only 55% people used tube-well water in the 1980s and this increased to 95% in the mid-1990s.On the otherhand, many people have changed their tube-well [24], [25].Therefore it is needed other drinking exposure sources for constructing historical exposure that can truly estimate exposure. Time–weighted lifetime average (average) water exposure was calculated for each participant based on the different water sources used since their birth. An approximate time-weighted mean arsenic exposure level (µg/L) was calculated over the life time of each subject as Σ_j_ ((a_j_ c_j_)/Σ_j_a_j,_), where a_j_ is the number of years a well with arsenic concentration c_j_ was used. Cumulative water arsenic exposure means total concentration one participants can consume. The cumulative arsenic exposure (µg-years/L) was calculated as Σ_j_ (a_j_ c_j_), where a_j,_ is the number of years a well with arsenic concentration c_j_ was used [23], [26].

### Childhood Mortality Data

All childhood deaths (age 5–18 years) were ascertained for the period between 1 January 2003 to 31 December 2010. Causes of deaths were identified from routine VA conducted by specially trained field staff of HDSS who were unaware of the arsenic exposure of the household members. A close relative, namely the mother of the deceased, was interviewed using a structured verbal-autopsy questionnaire to captures signs and symptoms of diseases/conditions that were present prior to death and any medical consultations before death. Two physicians independently reviewed the VA questionnaire and assigned the underlying cause of death. In case of disagreement, a third physician resolved the cause of death. Assignment of causes of death was done in accordance with the verbal autopsy standards that have been developed by the INDEPTH network and the World Health Organization (WHO) [27]. The cause of death was coded following the 10^th^ revision of International Classification of Diseases (ICD-10) of the WHO.

### Analyses

Covariates (age, sex, educational attainment, socio-economic status) were derived from the HDSS database. Asset scores were calculated through principal component analysis [28] and categorized into quintiles: lowest as poorest and highest as richest. Those with missing SES information (N = 1039) were imputed by the series mean.

The mortality risk of arsenic exposures was estimated by Cox’s proportional hazard models, adjusting for potential confounders. First we assessed crude association and then adjusted for covariates. A covariate was identified as a potential confounder if associated with exposure and outcome at p≤0.10 significance level. Potential confounders that were found to change the effect estimates by 5% or more were included in the adjusted multivariate models. We included sex in the model as a priori Baseline and time-weighted lifetime average lifetime exposure were divided into five groups (<10, 10.0–50.0, 50.1–150.0, 150.1–300.0, and more than 300.0 µg/L, respectively). Cumulative arsenic exposure or concentration-time product exposure to arsenic was divided into four groups <1000, 1000–4000, and >4000 µg-yrs/L. Since there were fewer cancer and cardiovascular deaths, baseline exposure was divided into four groups (<10, 10.0–50.0, 50.1–150.0, and more than 150.0, respectively). For all analyses, lowest exposure category was used as reference. Adjusting for sex, SES, education, and baseline age, we plotted cumulative hazard functions for each exposure category. Each analysis was performed separately for each exposure (baseline, time-weighted lifetime average and cumulative). All analyses were conducted in SPSS 15.0 (SPSS Inc, Chicago, Illinois).

## Results

There were 166,934 individuals of age 4 years and above were interviewed and examined by the field staff during the arsenic baseline study (2002–2003) who were available and met the AsMat criteria. Of them, a total of 58,406 participants were assembled in our cohort who met our eligibility criteria (age 5–18 years) from the previous baseline study. During 2003–2010, 283 deaths were ascertained and 23,429 individuals were lost to follow-up mainly due to migration-out for new job, marriage, or education.

The mean baseline age was 11.7±3.5 years. There was no age difference between boys and girls (11.6±3.5 *vs.* 11.78±3.5). The total follow-up time was 381,101 person-years. **Table 1** shows the distribution of demographic and exposure characteristics for the baseline cohort and deceased individuals. Girls had slightly higher mortality than boys (4.7 *vs.* 5.0 per 10000 person-years, ) and adolescents had double the death rate of younger children (6 *vs*. 4 per 10000 person-years, *P*<0.001). The crude death rate was much higher among the “no-education” group compared to “education” groups” (*P*<0.001).

**Table 1:** Variation in arsenic exposure and mortality for selected characteristics of childhood participants.

	Average As in well water (µg/L)		Baseline As in well water (µg/L)		Baseline Cohort (n = 58406)		Death (n = 185)		Rate *
	Mean	SD	Mean	SD	N	%	N	%	
*Sex*					(χ2 = 0.2,1 P = 0.64)				
Male	177.66	152.27	119.08	172.88	28756	49.23	88	47.57	4.68
Female	178.10	147.65	114.57	168.69	29650	50.77	97	52.43	5.03
*Age in years*					(χ2 = 4.8, P = 0.017)				
5–11	175.42	153.92	117.81	169.91	27730	47.48	73	39.46	3.67
12–18	180.12	146.22	115.86	171.56	30676	52.52	112	60.54	6.15
*Education category*					(χ2 = 209.0, P = 0.000)				
0	167.06	150.40	118.39	179.10	7970	13.65	92	49.73	20.28
1–5	178.78	150.87	118.65	170.38	31102	53.25	68	36.76	3.21
6+	180.90	148.04	113.14	167.84	19334	33.10	25	13.51	2.02
*SES*					(χ2 = 16.2, P = 0.003)				
1 poorest)	167.59	149.95	121.54	181.75	10014	17.15	45	24.32	6.97
2 (poor)	175.01	150.77	118.54	169.29	11655	19.96	42	22.70	5.52
3	180.71	151.11	120.50	170.52	13312	22.79	48	25.95	5.72
4 (rich)	189.54	151.11	118.15	168.52	12510	21.42	22	11.89	2.63
5 (richest)	173.60	145.26	104.48	164.20	10915	18.69	28	15.14	3.84
*Baseline arsenic exposure in well water*					(χ2 = 5.9, P = 0.20)				
<10	123.98	133.87	1.42	2.04	28901	49.48	83	44.86	4.39
10–49	128.09	131.75	24.84	11.13	5092	8.72	15	8.11	4.47
50–149	148.26	93.18	99.91	29.52	5634	9.65	13	7.03	3.52
150–299	216.77	81.93	222.10	41.58	10329	17.68	39	21.08	5.77
300+	364.49	144.79	449.34	154.44	8450	14.47	35	18.92	6.48
*Time-weighted lifetime average arsenic exposure*					(χ2 = 3.8, P = 0.43)				
<10	1.46	2.41	2.44	11.03	8527	14.60	24	12.97	4.21
10–49	28.93	12.04	21.27	56.38	5648	9.67	17	9.19	4.61
50–149	101.08	29.61	59.73	91.45	13357	22.87	44	23.78	5.03
150–299	216.80	41.73	142.85	139.98	19946	34.15	56	30.27	4.33
300+	415.38	117.02	277.56	249.35	10928	18.71	44	23.78	6.27
*Cumulative arsenic exposure*					(χ2 = 6.46, P = 0.04)				
<1000	30.60	35.92	19.60	53.92	19274	33.00	54	29.19	4.16
1000–40000	201.55	82.80	132.41	145.86	29558	50.61	88	47.57	4.53
>4000	401.34	134.37	264.23	254.73	9574	16.39	43	23.24	7.54

Crude death rate per 10000 person-years.

**Figure 1:**
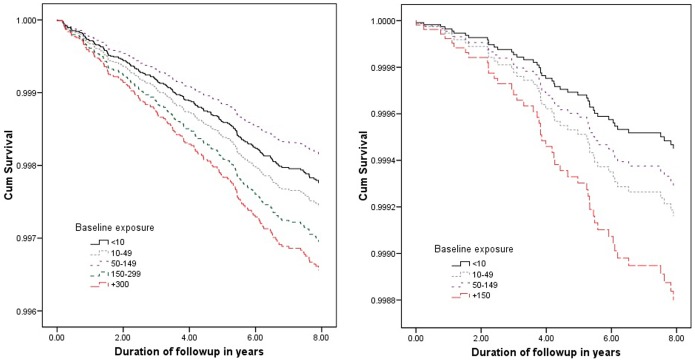
Cumulative survival function of all-cause, cancer and cardiovascular mortality plotted against time for baseline arsenic exposure categories.

Arsenic exposures (baseline, time-weighted lifetime average and cumulative exposure) were associated with increased all-cause childhood mortality (**Table 2**). The average arsenic exposure was associated with all-cause childhood deaths in a dose-dependent manner with an adjusted HR 1.88 (95% CI, 1.14–3.10) for the highest average exposure category. However, dose-dependent relationship was not monotonic. The risk of death due to all-cause and to cancer, cardiovascular diseases, increased with increasing exposure (**Figure 1**).

**Table 2:** Hazard ratio (HR) for all-cause mortality in childhood participants in relation to baseline, cumulative and time-weighted lifetime average arsenic exposure.

Exposure variables	Baseline participant	Person-years	Deaths	Crude HR (95% CI)	Adjusted HR† (95% CI)
Baseline arsenic in well water					
<10	28901	189047	83	1.0	1.0
10–50	5092	33549	15	1.02 (0.59–1.76)	1.13 (0.65–1.96)
51–150	5634	36918	13	0.80 (0.45–1.44)	0.81 (0.45–1.46)
151–300	10329	67591	39	1.31 (0.90–1.92)	1.35 (0.92–1.97)
>300	8450	53995	35	1.48 (0.99–2.19)	1.51 (1.01–2.23)
P for trend, *P*<0.05Time-weighted lifetime average arsenic in well water					
<10	8527	57073	24	1.0	1.0
10–50	5648	36883	17	1.09 (0.59–2.04)	1.37 (0.74–2.57)
51–150	13357	87520	44	1.19 (0.73–1.96)	1.44 (0.88–2.38)
151–300	19946	129469	56	1.03 (0.64–1.66)	1.22 (0.75–1.98)
>300	10928	70155	44	1.49 (0.91–2.45)	1.88 (1.14–3.10)
P for trend, *P*<0.5Cumulative arsenic in well water					
<1000	19274	129798	54	1.0	1.0
1000–4000	29558	194283	88	1.09 (0.78–1.53)	1.17 (0.84–1.65)
>4000	9574	57020	43	1.80 (1.21–2.69)	1.90 (1.25–2.89)
P for trend, *P*<0.05					

† Adjusted with baseline age, educational attainment, SES.

**Figure 2:**
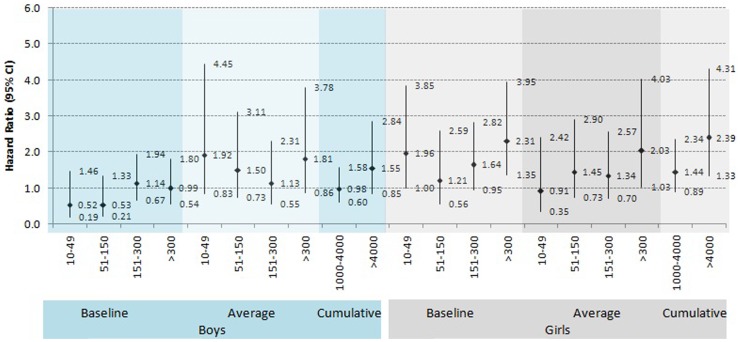
Age, education attainment, SES adjusted Hazard ratios for male and female by baseline, time-weighted lifetime average and cumulative exposure categories.

Similar significant risk of trend was observed in baseline (*P* for trend = 0.054) and cumulative exposure categories (*P* for trend = 0.034). Stratifying by gender, girls had higher risk of death comparing to boys (**Figure S1,** **Figure 2**). Multivariable adjusted HRs for girls were 2.31 (95% CI 1.35–3.95); for boys 1.00 (95% CI 0.54–1.80) compared to baseline arsenic exposure categories >300 µg/L, respectively. Similar significant risk was observed in baseline and cumulative exposure categories for gender differences and overall all mortalities (*P* for trend = 0.0001).

The mortality pattern from childhood mortalities in relation to baseline arsenic concentration is shown in Table 3 (**Table S1**). Considering small numbers, the two highest exposure categories were merged. The crude death rate per 100,000 was 8.5, 11.9, 10.8, and 18.9 for exposure categories, <10, 10–50, 51–150, and >150, respectively. For all cancer and cardiovascular deaths, the increased risk was pronounced in relation to baseline arsenic concentration (**Table 3** **, Table S2**). There was a steep increase in crude death rate from the 3 lower categories to the highest exposure category, and the overall crude death rate was 12 per 100,000 individuals. The risk of childhood cancer and cardiovascular deaths was significant. Multivariable adjusted HRs amounted to 2.49 (95% CI 1.13–5.16) comparing ≥150.1, respectively (*P* for trend = 0.023) (data not shown). Although the numbers are very small, the wide confidence intervals included unity confirming that the relationship may become significant at all levels if the numbers are increased or expected latency period occurs.

**Table 3:** Hazard ratio (HR) for cancer and cardiovascular related mortality in childhood participants in relation to baseline arsenic exposure.

Exposure variables	Baseline participant	Person-years	Deaths	Rate*	HR (95% CI)	
Baseline arsenic in well water					Unadjusted	Adjusted†
<10	28853	189047	16	8.5	1.0	1.0
10–50	5083	33549	4	11.9	1.41 (0.47–4.21)	1.53 (0.51–4.57)
51–150	5629	36918	4	10.8	1.28 (0.43–3.83)	1.29 (0.43–3.87)
>150	18743	121586	22	18.9	2.14 (1.12–4.07)	2.18 (1.15–4.16)

Test for Trend, *P*<0.05,

* Mortality rates per 100,000 person-years,

† Adjusted with baseline age, sex education attainment and socioeconomic status.

## Discussion

In this large population-based prospective study of rural Bangladeshi children, we observed increased childhood mortality associated with increased As exposure via contaminated well water.

Few previous studies have focused on childhood cancer in low exposure settings but those studies used ecologic exposure assessment, and there was possibility of misclassification of outcome data [12], [13]. The issue of As exposure and childhood mortality has not been studied in any arsenic exposed areas as such Bangladesh. The results available from previous studies cannot be used to infer any causal relationship due to their ecologic design, rather they may be considered as hypothesis generating, and therefore merits further study to seek more definitive answer to confirm or refute. Thus, this is a first prospective cohort study with individual level As exposure exploring causal association. Earlier we reported excess adult deaths in Matlab population [6], whereby Matlab provides a unique opportunity to investigate these arsenic health effects, considering individual exposure assessments. Considering the magnitude of HR we identified, along with the consistency of findings in all exposure categories suggest that these findings are true, not due to chance or bias, although the number of overall childhood death cases was relatively very small contrary to the large exposed population. Furthermore, we also demonstrated that arsenic-induced epigenetic modifications in *utero* may potentially influence disease outcomes in later life [29]. Thus this is a biologically significant risk that merits public concern.

More importantly, we observed a gender difference on the effect of all-cause childhood deaths. Girls had higher risk of deaths compared to boys in all exposure categories. Nearly all epidemiological studies concluded that men have higher risk of developing all arsenic-induced negative effects [1], [4], [30]–[34] including our previous skin lesion study [18], except one [35]. Earlier we demonstrated in the Matlab population that arsenic readily crosses the placenta, therefore a positive correlation occurred between maternal and cord blood arsenic [36].

Secondly, the magnitude of the effect estimates we observed were large in relation to baseline exposure categories comparing relatively small number of childhood cancers and cardiovascular deaths that have been identified in this cohort. There has been some interest on the association of As exposure and childhood cancer mortalities in recent years using group-level aggregated exposure data, but we found a sharp increased risk of liver and leukemia cancer mortalities in this study, which could also be due to the increased fatality of cancers related to arsenic based on the evidence that arsenic causes increased mortality from many cancers including lung, liver, kidney, and bladder cancers and not just due to increased incidence alone [1]. This is also true in case of mortality from pulmonary TB as reported by Smith et al, 2011 [37]. Higher multivariable adjusted HRs were found for adolescents (HR = 2.46, 95% CI, 1.03–5.88) than children (HR = 1.31, 95% CI 0.42–4.09) (data not shown). The plausibility of a causal association between exposure and disease is enhanced if experimental treatment can produce a similar condition.

Strictly speaking, there have been only five childhood cancer studies [11]–[15] to compare with our results. All are ecologic in nature, and no study showed childhood cardiovascular or all-cause death risks. A non-significant relative risk 1.39 (CI: 0.7–2.76) for lymphoblastic leukemia was reported from a Canadian case-control study [14]. Another non-significant relative risk was reported for all cancer combined 1.25 (CI 0.91–1.69), and for leukemia 1.37 (CI: 0.92–1.83) in Nevada [13]. Similar results were observed in Chile for all cancers combined [12], except liver cancer mortality was markedly increased (RR = 10.6, 95% CI 2.9–39.2, *P*<0.001). Ecologic bias may have occurred in those studies based on aggregated group level exposure data causing measurement error. As such those studies may not be used as inferring a causal relationship rather used as hypothesis generating. Truly this is the first study that enabling us to analyze childhood mortalities. Major studies focused on adult carcinogenic risks, evidencing multisite carcinogenetic role and therefore its carcinogenicity among children remained uninvestigated. But the overall carcinogenicity process is still undiscovered, and theoretically arsenic causes aberrant cell proliferation including alteration in apoptosis. In combination of disrupt cell proliferation, genetic mutation, chromosomal breakage and genetic damage may enhance carcinogenesis in children [38]–[40]. However, children and adults may have differences in carcinogenic risks reflecting differences in their tissue dosages and thereby differences in sensitivity.

The major strength of our study included larger sample size, individual exposure data, assessment of different exposures by outcomes, and independent outcome data were collected prospectively from the HDSS databases. The mortality data set included relatively larger childhood mortality data over an extended follow-up time (over 185 deaths during more than 0.4 million person-years at risks). Without a death registry, proper case ascertainment may be uncertain in a developing country, but, standardized approaches from widely validated verbal autopsy methods attributed to the strength of the study. VA is a well-known instrument ascertaining the cause of death based on information obtained from close relatives through systematic retrospective questioning [41]. Moreover, it is stemmed from findings of earlier ecologic studies [11]–[15] that indicated increased childhood deaths due to arsenic exposure. Another strength of our study is that any biases were minimized by combining individual outcome data from the regular HDSS monthly surveillance at the household level and exposure data at the individual level.

Despite major strengths, limitations can be attributed to some unmeasured or imprecisely measured potential confounding factors. A major weakness of this study is lack of exposure history between 2003 to 2010. Change of drinking water sources of the cohort during this period is quite natural. Well water arsenic exposure is the only exposure parameter considered no other sources of exposure have been estimate.The unaccounted changes of As sources across the observational time is a weakness. Therefore, findings are to be interpreted under the assumption of constant source of As as measure in 2003 and that the impact of possible changes is unknown. Secondly 39% individuals were lost during the study period. Majority moved out (taking new job 35.4%, married 17.7%, education 12.3%, children follow parents 12.7%, others 37.4%). Moreover, lost to follow-up is a problem regardless of any possible assuring comparisons. We simply do not know how they could have affected HRs if they were not lost.

However, there were no significant difference in exposure distribution between included or excluded persons of this cohort (Table S3). Thirdly, drinking water was the reported primary source of exposure estimates. Arsenic exposure may also be contributed by food and other water sources [30], [42]. A study from Bangladesh has shown that rice often contains more than 100 µg/kg of arsenic in arsenic affected areas [43] and rice alone may contribute about 50 µg arsenic per day. However, recent study from Bangladesh demonstrated that it is often adequate to estimate an individual’s past arsenic exposure based on the reported main sources of drinking water and the influence of neighbouring water sources was limited [44]. Fourthly, we did not have pathological reports for case ascertainment, and we have no information on SES among 1039 individuals (3.9% of total cohort). We do not believe our study findings would differ much with exclusion of these factors. Moreover, there are no reasons to believe that misclassification of cause of death is associated with arsenic exposure levels. Not knowing the level of arsenic exposure indicates no bias while assigning cause of childhood deaths.

We also found marked increase in all cause childhood mortality (HRs = 1.53, 95% 1.03–2.28) among adolescents (age range 12–18) as well as liver cancer and leukemia deaths having relatively short latency. In addition, girls were at increased risk compared to boys. There have been many studies of effects of arsenic exposure in adults, but very little attention has been given to potential effects resulting from exposure to infants and children. It has become clear that the long-term effects of toxic substances in children need to be investigated and understood to protect children’s health, and later their health as adults.

Despite some limitation, this study was feasible because of the uniqueness of the Matlab population, which includes individual level As exposure data and the independent prospective demographic surveillance system covering 0.2 million population for about five decades. Further studies are needed with a longer follow up period to investigate if the risk is further enhanced.

## Supporting Information

Figure S1 **Cumulative survival function of all-cause, cancer and cardiovascular related mortality in childhood participants’ mortality plotted against time for time-weighted lifetime average (average) and cumulative arsenic exposure categories.**
(TIF)

Table S1 **Selected characteristics of childhood cardiovascular and cancer death participants in relation to baseline arsenic exposure.**
(DOC)

Table S2 **Causes of child and adolescent mortality in relation with different exposures.**
(DOCX)

Table S3 **Comparison of the distribution of included mortality cases and survivors, as well as loss to follow-up cases.**
(DOCX)
