# Supplementary figures and images for: Increased Childhood Mortality and Arsenic in Drinking Water in Matlab, Bangladesh: A Population-Based Cohort Study
Source: PLoS One. 2013 Jan 28;8(1):e55014. doi: 10.1371/journal.pone.0055014 (PMC3557245; doi:10.1371/journal.pone.0055014)

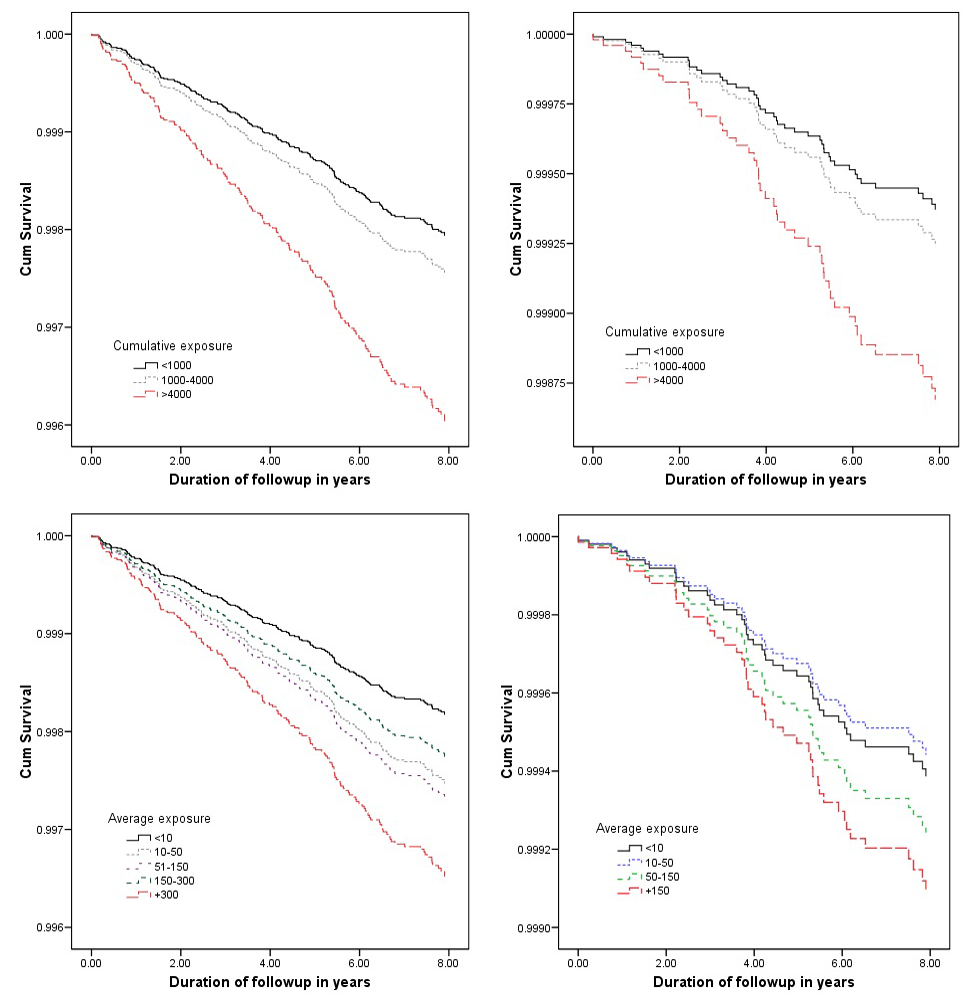

Supplement: Figure S1 — Cumulative survival function of all-cause, cancer and cardiovascular related mortality in childhood participants’ mortality plotted against time for time-weighted lifetime average (average) and cumulative arsenic exposure categories. (TIF) [file pone.0055014.s001.tif]
