# Supplementary material for: Increased Childhood Mortality and Arsenic in Drinking Water in Matlab, Bangladesh: A Population-Based Cohort Study
Source: PLoS One. 2013 Jan 28;8(1):e55014. doi: 10.1371/journal.pone.0055014 (PMC3557245; doi:10.1371/journal.pone.0055014)
Supplement: Table S1 — Selected characteristics of childhood cardiovascular and cancer death participants in relation to baseline arsenic exposure. (DOC) [file pone.0055014.s002.doc]

**Table S1.** Selected characteristics of childhood cardiovascular and cancer death participants in relation to by baseline arsenic exposure

| Baseline arsenic | As in well water (µg/L) | | Baseline Cohort (n=58406) | | Death (n=46) | | Rate* |
| --- | --- | --- | --- | --- | --- | --- | --- |
|  | Mean | SD | N | % | N | % |  |
| <10 | 4.08 | 1.39 | 28901 | 49.5% | 10 | 27.8% | 8.5 |
| 10-49 | 38.90 | 23.26 | 5092 | 8.7% | 4 | 11.1% | 11.9 |
| 50-149 | 144.81 | 107.32 | 5634 | 9.6% | 4 | 11.1% | 10.8 |
| >150 | 496.00 | 293.37 | 18779 | 32.2% | 18 | 50.0% | 18.9 |

*Crude death rate (100000) person-years
