# Supplementary material for: Increased Childhood Mortality and Arsenic in Drinking Water in Matlab, Bangladesh: A Population-Based Cohort Study
Source: PLoS One. 2013 Jan 28;8(1):e55014. doi: 10.1371/journal.pone.0055014 (PMC3557245; doi:10.1371/journal.pone.0055014)
Supplement: Table S2 — Causes of child and adolescent mortality in relation with different exposures. (DOCX) [file pone.0055014.s003.docx]

**Table S2.** Causes of child and adolescent mortality in relation with different exposures

| Cause of death | Number of Deaths | Age at baseline | | Baseline As in well water µg/L | | Average arsenic in Well Water (µg/L) | | Cumulative arsenic in well water (µg-yrs/L) | | Total year of exposure | |
| --- | --- | --- | --- | --- | --- | --- | --- | --- | --- | --- | --- |
|  | N | Mean | SD | Mean | SD | Mean | SD | Mean | SD | Mean | SD |
| All Cancers | 26 | 11.3 | 3.8 | 146.7 | 158.5 | 153.69 | 129.1 | 1890.9 | 1633.7 | 12.0 | 3.9 |
| All CVS | 20 | 13.1 | 3.2 | 165.2 | 162.2 | 235.4 | 154.2 | 3253.1 | 2110.1 | 10.7 | 3.5 |
| Endocrine | 7 | 9.9 | 3.6 | 169.4 | 190.7 | 186.1 | 174.5 | 2135.4 | 1900.5 | 9.2 | 2.1 |
| Respiratory | 4 | 14.1 | 1.6 | 167.0 | 43.5 | 180.0 | 64.4 | 2702.5 | 1046.8 | 15.0 | 1.3 |
| All other | 128 | 12.3 | 3.3 | 113.8 | 152.4 | 168.4 | 142.1 | 2231.9 | 1789.9 | 13.1 | 3.3 |
