# Supplementary material for: Increased Childhood Mortality and Arsenic in Drinking Water in Matlab, Bangladesh: A Population-Based Cohort Study
Source: PLoS One. 2013 Jan 28;8(1):e55014. doi: 10.1371/journal.pone.0055014 (PMC3557245; doi:10.1371/journal.pone.0055014)
Supplement: Table S3 — Comparison of the distribution of included mortality cases and survivors, as well as loss to follow-up cases. (DOCX) [file pone.0055014.s004.docx]

**Table S3.** Comparison of the distribution of included mortality cases and survivors, as well as loss to follow-up cases

| **Characteristics** | **Variables** | **Participated** | | **Loss to follow-up** | |
| --- | --- | --- | --- | --- | --- |
| Age | | N | % | N | % |
|  | 5-11 | 20494 | 58.8% | 7205 | 30.8% |
|  | 12-18 | 14385 | 41.2% | 16224 | 69.2% |
| Sex | |  |  |  |  |
|  | Male | 17285 | 49.6% | 11419 | 48.7% |
|  | Female | 17594 | 50.4% | 12010 | 51.3% |
| SES | |  |  |  |  |
|  | Poor 1 | 5824 | 16.7% | 4170 | 17.8% |
|  | 2 | 6984 | 20.0% | 4654 | 19.9% |
|  | 3 | 7748 | 22.2% | 5537 | 23.6% |
|  | 4 | 7710 | 22.1% | 4779 | 20.4% |
|  | Rich 5 | 6613 | 19.0% | 4289 | 18.3% |
| Education | |  |  |  |  |
|  | No | 4421 | 12.7% | 3512 | 15.0% |
|  | 1-5 | 20534 | 58.9% | 10530 | 44.9% |
|  | +6 | 9924 | 28.5% | 9387 | 40.1% |
| Baseline arsenic in Well water (μg/L) | | |  |  |  |
|  | <10 | 17298 | 49.6% | 11555 | 49.3% |
|  | 10-49 | 3092 | 8.9% | 1991 | 8.5% |
|  | 50-149 | 3410 | 9.8% | 2219 | 9.5% |
|  | 150-299 | 6222 | 17.8% | 4084 | 17.4% |
|  | 300+ | 4857 | 13.9% | 3580 | 15.3% |
| Average arsenic in Well water (μg/L) | | |  |  |  |
|  | <10 | 5469 | 15.7% | 3045 | 13.0% |
|  | 10-49 | 3393 | 9.7% | 2245 | 9.6% |
|  | 50-149 | 8095 | 23.2% | 5239 | 22.4% |
|  | 150-299 | 11663 | 33.4% | 8245 | 35.2% |
|  | 300+ | 6259 | 17.9% | 4655 | 19.9% |
| Cumulative arsenic in Well water (μg-years/L) | | |  |  |  |
|  | <1000 | 12522 | 35.9% | 6720 | 28.7% |
|  | 1000-4000 | 17760 | 50.9% | 11746 | 50.1% |
|  | >4000 | 4597 | 13.2% | 4963 | 21.2% |
